# Supplementary material for: Prediction of local failure after stereotactic radiotherapy in melanoma brain metastases using ensemble learning on clinical, dosimetric, and radiomic data
Source: Radiat Oncol. 2026 Mar 21;21:50. doi: 10.1186/s13014-026-02825-w (PMC13063852; doi:10.1186/s13014-026-02825-w)
Supplement: Supplementary file 1 — Supplementary Material 1 [file 13014_2026_2825_MOESM1_ESM.docx]

**Supplementary Table I**

| **Parameter** | **Value** |
| --- | --- |
| Manufacturer | Philips Health Systems (Eindhoven, The Netherlands); Siemens (Erlangen, Germany) |
| Scanner Models | Philips: Achieva, Achieva dStream, Ingenia  Siemens: Aera, Avanto, Avanto_fit, Espree, MAGNETOM, Prisma, Symphony, TrioTim, Verio |
| Field Strength | 1.5 / 3 Tesla |
| Sequence Type | 3D T1-weighted MPRAGE |
| Matrix | 240 × 240 – 256 × 256 |
| Repetition Time (TR) | 587 – 2500 ms |
| Echo Time (TE) | 2.15 – 35 ms |
| Flip Angle (FA) | 7 – 80° |
| Voxel Size (isotropic) | 0.23 - 1 mm³ |
| Slice Thickness | 1 mm |

**Supplementary Material I**

A prerequisite for a robust radiomics model is, that the RFs are not affected by inter-observer variability of the contours. However, since only one expert contour per lesion was available, three additional artificial contour datasets to simulate additional observers were created. One artificial contour datasets was created by facilitating the nnUnet trained on 57 MR images and corresponding expert contours from Center I. The network was tested on 20 other patients with a total of 58 lesions. The network detected 52 lesions which corresponds to a sensitivity of 90%. A comparison of the detected lesions with the expert contours yielded a median Sørensen-Dice coefficient of 0.82. The other two artificial contour datasets were made by applying either a morphological erosion, morphological dilation or identity function to each of the expert contours. Which of these three function was used for a certain contour was random.

**Supplementary Material II**

RF were calculated using Pyradiomics v3.0.1 with a fixed bin count of 32 for the original and the wavelet filtered images. For the remaining parameters the default values were used to calculated features of the following feature classes: shape, firstorder, glcm (grey-level co-occurrence matrix), glrlm (grey-level run-length matrix), glszm (grey-level size zone matrix), gldm (grey-level dependence matrix).

To reduce the risk of overfitting the number of features was reduced by the following steps:

1. Contour dependency: Only features with similar distributions (see statistical details) for all four contour sets were retained.

2. Univariate analysis: First, all features that did not meet the assumptions of the Cox Proportional Hazards Model (CPHM) or had zero variance were eliminated. Second, a univariate CPHM was trained for each feature on the training set. Features that did not have a model coefficient significantly different from zero were excluded.

3. Feature clustering: A hierarchical tree with 1−Spearman correlation coefficient as distance and complete linkage was constructed and cut at a height of 0.2. From each cluster, the RF with the lowest p-value according to the univariate CPHM from step 2 was retained.
